# The next generation of immunotherapy: keeping lung cancer in check

**DOI:** 10.1186/s13045-017-0456-5

**Published:** 2017-04-24

**Authors:** Ashwin Somasundaram, Timothy F. Burns

**Affiliations:** 1 University of Pittsburgh Medical Center, Pittsburgh, PA USA; 2 Division of Hematology-Oncology, Department of Medicine, University of Pittsburgh, Hillman Cancer Center Research Pavilion, 5117 Centre Avenue, Pittsburgh, PA 15213-1863 USA

**Keywords:** Non-small cell lung cancer, Tumor-infiltrating lymphocytes, Regulatory T cells, Programmed death 1, Programmed death ligand 1, Cytotoxic T-lymphocyte-associated protein 4

## Abstract

Lung cancer is the deadliest malignancy with more cancer deaths per year than the next three cancers combined. Despite remarkable advances in targeted therapy, advanced lung cancer patients have not experienced a significant improvement in mortality. Lung cancer has been shown to be immunogenic and responsive to checkpoint blockade therapy. Checkpoint signals such as CTLA-4 and PD-1/PD-L1 dampen T cell activation and allow tumors to escape the adaptive immune response. Response rates in patients with pretreated, advanced NSCLC were much higher and more durable with PD-1 blockade therapy compared to standard-of-care, cytotoxic chemotherapy. Therefore, PD-1 inhibitors such as nivolumab and pembrolizumab were rapidly approved for both squamous and nonsquamous lung cancer in the pretreated population. The advent of these new therapies have revolutionized the treatment of lung cancer; however, the majority of NSCLC patients still do not respond to PD-1/PD-L1 inhibition leaving an unmet need for a large and growing population.

Immunotherapy combinations with chemotherapy, radiation therapy, or novel immunomodulatory agents are currently being examined with the hope of achieving higher response rates and improving overall survival rate. Chemotherapy and radiation therapy has been theorized to increase the release of tumor antigen leading to increased responses with immunotherapy. However, cytotoxic chemotherapy and radiation therapy may also destroy actively proliferating T cells. The correct combination and order of therapy is under investigation. The majority of patients who do respond to immunotherapy have a durable response attributed to the effect of adaptive immune system’s memory. Unfortunately, some patients’ tumors do progress afterward and investigation of checkpoint blockade resistance is still nascent.

This review will summarize the latest efficacy and safety data for early and advanced NSCLC in both the treatment-naïve and pretreated settings. The emerging role of immunotherapy for the treatment of small cell lung cancer and malignant mesothelioma will also be discussed.

## Background

There will be an estimated 224,390 new cases of lung cancer, and an estimated 158,080 lung cancer deaths in 2016. Despite many advances in treatment, the 5-year overall survival for advanced lung cancer is still dismal [1]. Immunotherapy has redefined standard-of-care treatment in the second line, and more recently in the first-line settings, but long-term survival data is still too immature to determine its overall impact in the prognosis of lung cancer [2]. For many years, lung cancer had been thought not immunogenic. However, elevated levels of cytotoxic T-lymphocyte-associated protein 4 (CTLA-4), programmed death 1 (PD-1)/programmed death ligand 1 (PD-L1), B7-H3, and B7-H4 on CD8+ tumor-infiltrating lymphocytes (TILs) have been shown in NSCLC [3]. These findings suggest that the immune system plays a much larger role in the control of lung cancer than previously thought.

**Fig. 1:**
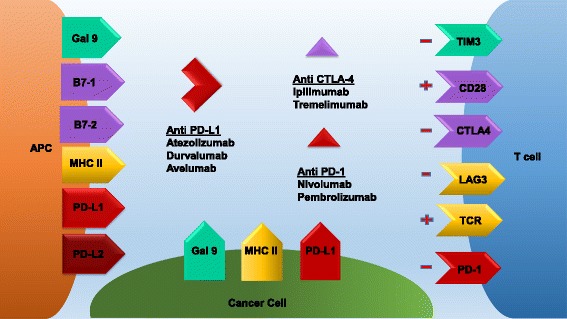
Multiple receptor-ligand interactions determine T cell response. Binding of the TCR to the APC MHC II receptor presenting antigen leads to antigen specific T cell proliferation and activation. Conversely, LAG-3 binding to the APC MHC can interfere with this process. Furthermore, PD-1 on T cells binding to PD-L1 on APCs, tumor cells, or Tregs can also dampen this response. Nivolumab and pembrolizumab inhibit PD-1, while atezolizumab, durvalumab, and avelumab inhibit PD-L1 leading to T cell activation. B7-1 or B7-2 binding to CD28 also leads to T cell activation. However, if CTLA-4 binds to B7-1 or B7-2 instead, this response is halted. Ipilimumab and tremelimumab inhibit CTLA-4 allowing for T cell activation

The adaptive immune system prevents and controls tumor growth in part through T cell activation. Cancer cells release tumor antigen which is recognized as foreign by antigen presenting cells (APCs) or dendritic cells (DCs). Once APCs verify the tumor antigen as foreign via their interaction with CD4+ and CD8+ T cells, the APCs signal for the proliferation of various T cell subtypes that also recognize the tumor antigen. Many of these lymphocytes become CD8+ cytotoxic T cells that infiltrate the tumor as tumor-infiltrating lymphocytes (TILs), but some become regulatory or suppressor T cells (Tregs). The normal function of Tregs is to induce several signaling checkpoints that hamper this process of T cell activation in order to prevent a toxic, autoimmune, positive feedback loop. However, the tumor microenvironment takes advantage of these checkpoint signals in an effort to prevent an anti-tumor T cell response. Examples of these inhibitory signals that have FDA-approved agents targeting them include the CTLA-4 and PD-1/PD-L1 receptors. Antibodies targeting CTLA-4 were initially approved for checkpoint blockade in melanoma but were unfortunately ineffective in NSCLC [4]. Preclinical studies demonstrated that PD-L1 expression correlates with TILs and immunogenicity in NSCLC, which suggested that NSCLC may respond to anti-PD-1/anti-PD-L1 therapy [5]. Furthermore, early safety data studies demonstrated promising efficacy of PD-1 inhibition in NSCLC [6], and antibodies targeting PD-1 have shown remarkable activity in lung cancer as well as melanoma [7] (Fig. 1).

Checkpoint inhibitors, specifically PD-1/PD-L1 inhibition therapy, have been approved in the treatment of certain subgroups of patients with advanced NSCLC. Landmark trials have shown greater overall survival and higher responses to checkpoint inhibition in NSCLC compared to standard-of-care first line and second line chemotherapy along with greater durability of response in a subset of patients. However, still many patients do not respond to these agents. Therefore, new combinations and further evaluation of immunotherapy will be necessary.

## Clinical experience with anti-PD-1/anti-PDL-1 agents in lung cancer

### Pretreated metastatic NSCLC

#### Anti-PD-1 agents

Early phase studies showed promising safety and response with nivolumab in advanced NSCLC, leading to the development of phase 2/3 studies [8] (Table 1). The phase 3 CheckMate 017 trial compared the anti-PD-1 agent, nivolumab, to docetaxel in patients with advanced pretreated, squamous (SQ) NSCLC, and this study lead to the first FDA approval for immunotherapy in NSCLC. Patients experienced a longer median overall survival (OS) with nivolumab (9.2 months, 95% CI 7.3–13.3) versus previous standard-of-care chemotherapy: docetaxel (6.0 months, 95% CI 5.1–7.3) (HR = 0.59, 95% CI 0.44–0.79, *p* = 0.00025). One-year OS was also higher with nivolumab (42%, 95% CI 34–50) compared to docetaxel (24%, 95% CI 17–31). Median duration of response (DOR) was higher with nivolumab (not reached, 95% CI 2.9–20.5+) compared to docetaxel (8.4 (1.4+–15.2+)). Median progression-free survival (PFS) was longer with nivolumab (3.5 months, 95% CI 2.1–4.9) compared to docetaxel (2.8 months, 95% CI 2.1–3.5) (HR = 0.62, 95% CI 0.47–0.81, *p* = 0.0004). This was reflected in a 1-year PFS that was higher with nivolumab (21%, 95% CI 14–28) compared to docetaxel (6%, 95% CI 3–12). Finally, median overall response rate (ORR) was higher with nivolumab compared to docetaxel (20 versus 9%, *p* = 0.0083). Interestingly, although PD-L1 positivity was not a requirement, patients with PD-L1+ tumors showed improved efficacy with nivolumab. However, patients with PD-L1 negative squamous tumors still benefit more from nivolumab compared to docetaxel. Grade 3–4 treatment-related adverse events (TrAEs) occurred in 7% of patients with nivolumab and 55% of patients treated with docetaxel along with three docetaxel-related deaths [9].

**Table 1:** Selected completed immunotherapy trials in pretreated, advanced NSCLC

Trial name	Phase	Histology	Therapy	mOS	1-year OS (%)	DOR	mPFS	1-year PFS (%)	mORR (%)	Ref
CheckMate 017	3	SQ	NIVO	9.2	42	NR	3.5	21	20	[9]
			DOC	6	24	8.4	2.8	6	9	
CheckMate 057	3	NONSQ	NIVO	12.2	50.5	17.1	NR	18.5	19.2	[10]
			DOC	9.4	39	5.6	NR	8.1	12.4	
KEYNOTE-010	2/3	ALL	PEMBRO 2 (PD-L1+)	9.4 (14.9)	NR	NR	3.9	NR	NR	[12]
			PEMBRO 10 (PD-L1+)	10.8 (17.3)	NR	NR	4	NR	NR	
			DOC	8.6 (8.2)	NR	NR	4	NR	NR	
POPLAR	2	ALL	ATEZO	12.6	NR	NR	4.2	NR	17	[13]
			DOC	9.7	NR	NR	NR	NR	NR	
OAK	3	ALL	ATEZO	13.8	55	16.3	2.8	NR	14	[15]
			DOC	9.6	41	6.2	4.0	NR	13	
KEYNOTE-021	1/2	ALL	PEMBRO + IPI	17	NR	14	6	NR	24	[17]

*NR* not reached

The phase 3 CheckMate 057 trial examined nivolumab to docetaxel in patients with advanced pretreated, nonsquamous (NONSQ) NSCLC. Median OS was again longer with nivolumab (12.2 months, 95% CI 9.7–15.0) compared to docetaxel (9.4 months, 95% CI 8.0–10.7) (HR = 0.73; 95% CI, 0.59–0.89; *p* = 0.00155). One-year OS was higher with nivolumab (50.5%, 95% CI 44.6–56.1) compared to docetaxel (39.0%, 95% CI 33.3–44.6) as well. Median ORR was higher with nivolumab compared to docetaxel (19.2 versus 12.4%; *p* = 0.0235; HR = 0.92, 95% CI 0.77–1.11, *p* = 0.393). In addition, DOR and 1-year PFS were also improved. Interestingly, PD-L1 negative, nonsquamous NSCLC did not show a significant benefit of immunotherapy over chemotherapy that was seen in the squamous population (<1% PD-L1 OS HR is 0.9 [95% CI 0.66–1.24], <5% PD-L1 OS HR is 1.01 [95% CI 0.76–1.33], and <10% PD-L1 OS HR is 1.00 [95% CI 0.76–1.31]). Grade 3–5 TrAEs occurred in 10.5% with nivolumab and occurred in 53.7% with docetaxel (also one docetaxel-related death) [10].

Early phase 1/2 data from KEYNOTE-001 showed promising safety and efficacy with pembrolizumab in PD-L1 positive, advanced NSCLC patients. This biomarker-driven study used its own companion diagnostic and led to the development of phase 2/3 studies [11] (Table 1). The KEYNOTE-010 trial was a phase 2/3 trial that compared pembrolizumab to docetaxel in pretreated, PD-L1+ (>1%) NSCLC patients and lead to the FDA approval of pembrolizumab with a companion PD-L1 diagnostic [12]. Median OS was 10.4 months for pembrolizumab 2 mg/kg and 12.7 months for pembrolizumab 10 mg/kg versus 8.5 months with docetaxel. Overall survival with pembrolizumab versus docetaxel favored pembrolizumab (HR = 0.71, 95% CI 0.58–0.88; *p* = 0.0008 for pembrolizumab 2 mg/kg and HR = 0.61, 95% CI 0.49–0.75; *p* < 0.0001 for pembrolizumab 10 mg/kg). However, median PFS was largely similar for pembrolizumab 2 mg/kg, pembrolizumab 10 mg/kg, and docetaxel (3.9, 4.0, and 4.0 months, respectively). Patients with tumors expressing at least 50% PD-L1 expression had significantly longer OS with pembrolizumab 2 mg/kg versus docetaxel (median 14.9 versus 8.2 months; HR = 0.54, 95% CI 0.38–0.77; *p* = 0.0002) and with pembrolizumab 10 mg/kg versus docetaxel (median 17.3 versus 8.2 months; HR = 0.50, 95% CI 0.36–0.70; *p* < 0.0001). PFS was significantly longer in this patient population with pembrolizumab 2 mg/kg compared to docetaxel (median 5.0 versus 4.1 months; HR = 0.59, 95% CI 0.44–0.78; *p* = 0.0001) and pembrolizumab 10 mg/kg compared to docetaxel (median 5.2 versus 4.1 months; HR = 0.59, 95% CI 0.45–0.78; *p* < 0.0001). These studies suggested that the degree of PD-L1 positivity may correlate with response rate. TrAEs were less common with pembrolizumab 2 mg/kg (13%) and pembrolizumab 10 mg/kg (16%) compared to docetaxel (35%) [12].

#### Anti-PD-L1 agents

Atezolizumab is a newer checkpoint inhibitor that targets PD-L1 compared to the PD-1 inhibitors above. It interferes with the interaction between PD-L1 and PD-1 as well as PD-L1 and B7-1, but does not interfere with the interaction between PD-L2 and PD-1 which may have biologic and therapeutic implications (Fig. 1). The POPLAR trial, a phase 2 study evaluating atezolizumab compared to docetaxel in 277 patients with pretreated, advanced NSCLC, showed a longer median OS with atezolizumab (12.6 months, 95% CI 9.7–16.4) compared to docetaxel (9.7 months, 95% CI 8.6–12.0) (HR = 0.73, 95% CI 0.53–0.99). Increased PD-L1 expression was associated with an increased mOS (TC3 or IC3: HR = 0.49, 95% CI 0.22–1.07; *p* = 0.068; TC2/3 or IC2/3: HR = 0.54, 95% CI 0.33–0.89; *p* = 0.014; TC1/2/3 or IC1/2/3: HR = 0.59, 95% CI 0.40–0.85; *p* = 0.005. TC0 and IC0: HR = 1.04, 95% CI 0.62–1.75; *p* = 0.871). Eleven percent of patients was treated with atezolizumab versus 39% of patients treated with docetaxel grade 3–4 TrAEs, and one patient in the atezolizumab group versus three patients in the docetaxel group died from a TrAE [13]. Overall, atezolizumab had higher OS compared to docetaxel (HR = 0.69, 95% CI 0.52–0.92) across all histologies and PD-L1 subgroups in advanced pretreated NSCLC. Interestingly, longer OS was also seen in TC0/IC0 or low PD-L1 expression patients in the squamous NSCLC population [14]. These findings were confirmed in the subsequent OAK trial, a phase 3 study evaluating atezolizumab compared to docetaxel in 850 patients with pretreated, advanced NSCLC, which demonstrated a longer median OS with atezolizumab (13.8 months, 95% CI 11.8–15.7) compared to docetaxel (9.6 months, 95% CI 8.6–11.2) (HR = 0.73, 95% CI 0.62–0.87, *p* = 0.0003). Patients benefitted regardless of PD-L status and histology. Given these findings, atezolizumab received FDA approval in this setting [15].

#### Combination therapy

Anti-PD-1 agents in combination with ipilimumab are currently being examined in several studies. The Lung Master Protocol (Lung-MAP) or SWOG1400 is a phase 2/3 study evaluating the role of targeted therapies in SQ NSCLC. The Cancer Genome Atlas (TCGA) has detected gene mutations that are potentially amenable to targeted therapy in SQ NSCLC. The Lung MAP study contains multiple phase 2 sub-studies which patients are assigned to sub-studies based on the genetic alterations present in their tumors. Mutations are identified with next generation sequencing. This trial also contains a sub-study for patients who do not have a molecular “match” which tests the combination of nivolumab with ipilimumab compared to standard-of-care nivolumab. This study is ongoing but will hopefully identify novel targeted therapeutic strategies for pretreated SQ NSCLC as well as assess the value of the nivolumab and ipilimumab combination [16]. In addition, the KEYNOTE-021 study contains cohorts which evaluate pembrolizumab in combination with ipilimumab in pretreated, advanced NSCLC. Earlier studies suggested that the use of lower doses (pembrolizumab 2 mg/kg and ipilimumab 1 mg/kg) may be efficacious and reduce combination toxicity. Grade 3–5 TrAEs occurred in 24% of patients and included diarrhea, and one treatment-related death from pancreatitis. Early evaluation revealed an ORR of 24% and SD rate of 40%. Median DOR was 14 months, median PFS was 6 months, and median OS was 17 months [17] (Table 1).

## Treatment-naïve metastatic NSCLC

### Monotherapy

CheckMate 012 also evaluated first-line nivolumab monotherapy in advanced NSCLC and demonstrated ORR was 23%, with 67% of responses being ongoing (5.3+ to 25.8+ months). Median PFS was 3.6 months, 24-week PFS rate was 41% (95% CI, 27–54), and median OS was 19.4 months. Twelve-month and 18-month OS rates were 73% (95% CI, 59–83) and 57% (95% CI, 42–70). Grade 3 to 4 TrAEs occurred in 19% of patients with rash being the most common AE [18, 19]. CheckMate 026 was a phase 3, randomized, open-label study of nivolumab monotherapy versus investigator’s choice chemotherapy in patients with treatment-naïve, advanced, ≥5% PD-L1 NSCLC. Unfortunately, this trial did not meet its primary endpoint of PFS. This may be possibly due to patient selection as a cutoff of ≥5% PD-L1 expression was utilized compared to a ≥50% cutoff which was explored in the positive KEYNOTE-024 trial described below [20].

KEYNOTE-024 was a phase 3, randomized study evaluating pembrolizumab 200 mg every 3 weeks versus platinum-based chemotherapy in treatment-naive, advanced, ≥50% PD-L1+ NSCLC. Patients were stratified to ECOG status, histology, and region of the world, with PFS as a primary endpoint. Secondary endpoints include OS, ORR, and safety. Median PFS was 10.3 months (95% CI, 6.7 to NR) with pembrolizumab versus 6.0 months (95% CI, 4.2 to 6.2) with chemotherapy, and HR for disease progression or death was 0.50 (95% CI, 0.37 to 0.68; *p* < 0.001). Six-month OS was 80.2% with pembrolizumab versus 72.4% with chemotherapy group, and HR for death was 0.60 (95% CI, 0.41 to 0.89; *p* = 0.005). The response rate was 44.8% with pembrolizumab versus 27.8% with chemotherapy. The median duration of response was not reached (1.9+ to 14.5+ months) with pembrolizumab versus 6.3 months (2.1+ to 12.6+) with chemotherapy. Treatment-related adverse events of any grade were 73.4% with pembrolizumab versus 90.0% with chemotherapy, and grade 3+ TrAEs were 26.6% with pembrolizumab versus 53.3% with chemotherapy [21]. In contrast to CheckMate 026, KEYNOTE-024 met its primary endpoint and has established a new standard of care in the first-line setting for advanced NSCLC with >50% PD-L1 expression. This suggests that a PD-L1 expression cutoff of greater than 50% may be predictive of response in the first-line setting; however, further study is necessary to determine the role of PD-L1 expression for prognostication and prediction in other settings. Additional trials examining anti-PD-L1 agents such as durvalumab are currently ongoing including a completed phase 1/2 trial (See Table 3).

### Combination therapy

Immunotherapy is also undergoing active evaluation in the first-line setting for advanced NSCLC. Several trials have examined these PD-1/PD-L1 inhibitors in combination with chemotherapy or CTLA-4 inhibitors (Tables 2 and 3).

**Table 2:** Selected completed immunotherapy trials in treatment-naïve NSCLC

Completed trial name	Phase	Histology	Therapy	mORR (%)	<1% PD-L1 ORR	>1% PD-L1 ORR	>50% PD-L1 ORR	<1% PD-L1 PFS	>1% PD-L1 PFS	>50% PD-L1 PFS	Ref
CheckMate 012	1	ALL	NIVO3	23	14	28	50	6.6	3.5	8.4	[18, 19]
			NIVO3 + IPIQ12W	47	30	57	100	4.7	8.1	13.6	
			NIVO3 + IPIQ6W	39	0	57	86	2.4	10.6	NR	
Trial Name	Phase	Histology	Therapy	PFS (months)	6-month OS (%)	ORR (%)	DOR	TrAEs (%)	3 + TrAEs (%)		
KEYNOTE-024	3	≥50% PD-L1	PEMBROQ3W	10.3	80.2	44.8	NR	73.4	26.6		[21]
			PT-DC	6.0	72.4	27.8	6.3months	90.0	53.3		
Trial name	Phase	Histology	Therapy	PFS (months)	OS	ORR	DOR	TrAEs (%)	3 + TrAEs (%)		
CheckMate 026	3	≥5% PD-L1	NIVO3	4.2	pending	pending	pending	71	18		[60]
			PT-DC	5.9	pending	pending	pending	92	51

**Table 3:** Selected ongoing immunotherapy trials in treatment-naïve, advanced or early NSCLC

Ongoing trial name	Phase	Histology	Therapy	Setting	Endpoints (starting with primary)	Ref
KEYNOTE-021	1/2	ALL	PEMBRO + PT-DC	Advanced, treatment-naïve NSCLC	PFS, ORR, OS, DOR, safety	[17]
KEYNOTE-189	3	ALL	PT-DC alone versus PT-DC + PEMBRO	Advanced, treatment-naïve NSCLC	PFS, ORR, OS, DOR, safety	[24]
IMPower 130	3	ALL	PT-DC + ATEZO	Advanced, treatment-naïve NSCLC	PFS, ORR, OS, DOR, safety	[26]
IMPower 111	3	SQ	Gemcitabine/PT + ATEZO	Advanced, treatment-naïve NSCLC	PFS, ORR, OS, DOR, safety	[29]
IMPower 110	3	NONSQ	Pemetrexed/PT + ATEZO	Advanced, treatment-naïve NSCLC	PFS, ORR, OS, DOR, safety	[28]
IMPower 131	3	SQ	Abraxane/PT + ATEZO	Advanced, treatment-naïve NSCLC	PFS, ORR, OS, DOR, safety	[27]
IMPower 150	3	NONSQ	PT-DC/ATEZO	Advanced, treatment-naïve NSCLC	PFS, ORR, OS, DOR, safety	[25]
NEPTUNE	3	ALL	PT-DC alone versus durvalumab + tremelimumab	Advanced, treatment-naïve NSCLC	PFS, ORR, OS, DOR, safety	[30]
MYSTIC	3	ALL	Durvalumab + tremelimumab	Advanced, treatment-naïve NSCLC	PFS, ORR, OS, DOR, safety	[31]
PEARLS	1b/2-3a	ALL	PEMBRO versus placebo	Adjuvant NSCLC	DFS, OS, lung cancer specific survival	[32]
SAKK 16/14	2	ALL	Durvalumab	Adjuvant NSCLC	Event-free survival at 1 and 5 years, OS, ORR, safety	[33]
NCT02259621	2	ALL	NIVO	Neoadjuvant NSCLC	Safety	[34]
Lung-MAP	2/3	SQ	Biomarker-driven combination with durvalumab, nivolumab, ipilimumab, and chemotherapy or targeted therapy	Advanced, pretreated SQ NSCLC	PFS, ORR, OS, DOR, safety	[16]

CheckMate 012 was a phase 1b, multi-cohort study exploring the safety and efficacy of nivolumab monotherapy versus in combination with platinum-based doublet chemotherapy (PT-DC) for treatment-naïve, advanced NSCLC. Nivolumab was evaluated with cisplatin plus gemcitabine for squamous or cisplatin plus pemetrexed for nonsquamous or carboplatin plus paclitaxel for all histologies. ORR was 33% for nivolumab 10 mg/kg plus cisplatin-gemcitabine, 47% for nivolumab 10 mg/kg plus cisplatin-pemetrexed, 47% for nivolumab 10 mg/kg plus carboplatin-paclitaxel, and 43% for nivolumab 5 mg/kg plus carboplatin-paclitaxel. In addition, 2-year OS rates were 25, 33, 27, and 62%. Forty-five percent of patients experienced grade 3 or 4 TrAEs most significant for pneumonitis [18, 19]. This small study suggested that the combining chemotherapy with immunotherapy may be feasible and improve response rates.

CheckMate 012 also evaluated nivolumab alone or in combination with ipilimumab in treatment-naïve, advanced NSCLC patients. Ipilimumab was evaluated with an every 6-weeks-cohort and an every 12-weeks-cohort, and patients were evaluated based on the level of PD-L1 expression. ORR, median PFS, and 1-year OS increased with higher PD-L1 expression and increased frequency of ipilimumab in combination with nivolumab. The combination arm also had greater toxicity and more TrAEs [22, 23]. This trial suggested that this combination may have significant activity and is being examined in several additional trials including the LUNG master protocol in the second-line setting.

KEYNOTE-021, a phase 1/2 study, evaluated the safety and efficacy of pembrolizumab in combination with PT-DC in treatment-naïve NSCLC patients regardless of tumor histology. Patient subgroups include the addition of bevacizumab or pemetrexed in the nonsquamous population. One treatment-related death with pericardial effusion occurred in the nonsquamous patient subgroup being treated with bevacizumab [17]. Based on early safety data from KEYNOTE-021, a phase 3, randomized, double-blind study named KEYNOTE-189 is currently evaluating the safety and efficacy of PT-DC alone compared to PT-DC in combination with pembrolizumab in treatment-naïve, nonsquamous NSCLC patients (NCT02578680). Patients are stratified according to smoking status, platinum agent, and PD-L1 status. Primary endpoint will be PFS, and secondary endpoints include ORR, DOR, OS, and safety [24].

In addition, several phase 3 trials examining atezolizumab in combination with a variety of platinum doublets with and without bevacizumab are currently ongoing for all treatment-naïve, advanced NSCLC patients, including squamous NSCLC and nonsquamous NSCLC using PD-L1 positivity as a biomarker [25–29].

NEPTUNE is a phase 3 trial that will evaluate the anti-PD-L1 agent durvalumab in combination with tremelimumab (CTLA-4 inhibitor) versus standard chemotherapy in treatment-naive, advanced NSCLC patients. Primary endpoint will include OS, and secondary endpoints will include PD-L1 status outcomes, PFS, ORR, DOR, and pharmacokinetics (PK) [30]. MYSTIC is a phase 3 trial that will evaluate durvalumab in combination with tremelimumab and durvalumab monotherapy versus standard chemotherapy in treatment-naive, advanced NSCLC patients. Primary endpoint will include OS, and secondary endpoints will include PD-L1 status outcomes, PFS, ORR, DOR, and PK [31].

### Adjuvant and neoadjuvant NSCLC

The role of PD-1/PD-L1 inhibition has yet to be established in early stage NSCLC. As the cure rates for resected lung cancer still ranges between 40 and 70% depending on stage, there is clearly a need for improved adjuvant therapy. Pembrolizumab is being evaluated as adjuvant therapy in PD-L1 positive NSCLC patients. “Pembrolizumab (MK-3475) Versus Placebo for Patients With Early Stage NSCLC After Resection and Completion of Standard Adjuvant Therapy” (PEARLS) trial, an international (European Thoracic Oncology Platform/EORTC-ETOP), phase 3, triple-blinded, placebo-controlled, randomized (1:1) study evaluating pembrolizumab after surgery and standard chemotherapy with a primary endpoint of DFS is currently underway [32]. SAKK 16/14 is a phase 2, multi-center, single-arm trial evaluating durvalumab in the perioperative setting along with standard chemotherapy in resectable NSCLC regardless of histology and PD-L1 expression. Patients will continue the treatment following surgical resection. The primary endpoint is event-free survival at 12 months, and secondary endpoints include OS, ORR, down-staging, complete resection, recurrence pattern, and toxicity [33]. In addition, nivolumab is being evaluated in the neoadjuvant setting in early NSCLC. After surgical resection, standard adjuvant therapy is planned with a primary endpoint of safety and exploratory endpoints of pathologic response, tumor markers by flow cytometry, and immunohistochemistry (IHC). Remarkably, two of the first three patients demonstrated major pathologic and radiographic response and one complete response in a squamous tumor that had a brisk T cell response [34] (Table 3).

## Small cell lung cancer

Small cell lung cancer (SCLC) is noted to have a high somatic mutation burden, and association with tobacco use, making it a potential target for checkpoint immunotherapy. Furthermore, PD-L1 (7.3%), B7-H3 (64.9%), and B7-H4 (2.6%) are present in SCLC suggesting that immunotherapy agents alone or in combination may be effective in a subset of these patients [35].

Ipilimumab (10 mg/kg every 3 weeks) versus placebo was examined in combination with standard first-line chemotherapy in extensive stage SCLC in a large phase 3 trial (*n* = 1,132) (NCT01450761). Patients received four cycles of combination therapy followed by maintenance ipilimumab versus placebo every 12 weeks. The primary endpoint was OS, and median OS was 11.0 months versus 10.9 months (HR = 0.94, 95% CI, 0.81–1.09, *p* = 0.3775) for ipilimumab combination therapy versus placebo therapy. Median PFS was 4.6 versus 4.4 months (HR = 0.85, 95% CI, 0.75–0.97). Rate of discontinuation was higher with the ipilimumab arm at 18% and five treatment-related deaths versus 2% and two treatment-related deaths with the placebo arm. The ipilimumab arm had more frequent episodes of diarrhea, rash, and colitis [36].

CheckMate 032 was a phase 1/2, open-label, multi-center study evaluating nivolumab monotherapy along with nivolumab in combination with ipilimumab for pretreated, extensive SCLC. ORR was 10% in the nivolumab 3 mg/kg (nivo3) arm, 33% in the nivolumab 1 mg/kg plus ipilimumab 1 mg/kg (nivo1 + ipi1) arm, 23% in the nivo1 + ipi3 arm, and 19% in the nivo3 + ipi1 arm. Grade 3–4 TrAEs occurred at a rate of 13% with nivo3, 30% with nivo1 + ipi3, and 19% with nivo3 + ipi1. No patients treated with nivo1 + ipi1 had grade 3–4 TrAEs. The most common grade 3–4 TrAEs included elevations in lipase and diarrhea. Two patients treated with nivo1 + ipi3 died from grade 5 TrAEs of myasthenia gravis and worsening renal failure while one patient treated with nivo3 + ipi1 died from treatment-related pneumonitis [37]. This study suggested that immunotherapy may be effective in a subset of SCLC patients.

CheckMate 331 is an ongoing phase 3 trial evaluating nivolumab monotherapy for pretreated, advanced SCLC. To date, durable responses have been seen regardless of PD-L1 expression. The primary endpoint will include OS, and secondary endpoints will include PFS, ORR, and safety [36]. CheckMate 451 is a phase 3, randomized, double-blind study evaluating nivolumab monotherapy or in combination with ipilimumab versus placebo as maintenance therapy after platinum-based, first-line chemotherapy (PT-DC) in advanced SCLC. Primary endpoints include OS and PFS. This trial will aim to accrue estimated 810 patients [38].

KEYNOTE-028 is an ongoing phase 1b study evaluating pembrolizumab for patients with pretreated, advanced, PD-L1+ SCLC. Pembrolizumab 10 mg/kg will be given every 2 weeks for up to 2 years or until progression or toxicity. Primary endpoints will include safety and response. Of the initial 16 patients evaluated, 53% developed TrAEs with only 1/16 patients that developed grade 3 toxicity. Twenty-five percent of the patients had a partial response, and 7% had stable disease with 37% of patients with progressive disease. Thirty-one percent of the patients were not evaluated at the time of analysis. Responses were found to be durable for 16+ weeks [39].

In summary, further evaluation will be necessary to establish the role of checkpoint inhibition immunotherapy in SCLC; however, initial combination studies appear promising (see Table 4).

**Table 4:** Selected immunotherapy trials in advanced SCLC

Trial name	Phase	Therapy	mOS	1-year OS	DOR	mPFS	1-year PFS	mORR (%)	Ref
NCT01450761	3	Ipilimumab	11	NR	NR	4.6	NR	NR	[61]
		PT-DC	10.9	NR	NR	4.4	NR	NR	
CheckMate 032	1/2	NIVO3	NR	NR	NR	NR	NR	10	[37]
		NIVO1 + IPI1	NR	NR	NR	NR	NR	33	
		NIVO1 + IPI3	NR	NR	NR	NR	NR	23	
		NIVO3 + IPI1	NR	NR	NR	NR	NR	19	
Ongoing trial name	Phase	Therapy	Setting	Endpoints (starting with primary)					
CheckMate 331	3	NIVO	Advanced SCLC	OS, PFS, ORR					[36]
CheckMate 451	3	NIVO versus NIVO + IPI	Advanced SCLC	OS, PFS					[38]
KEYNOTE-028	1b	PEMBROQ2W	Advanced SCLC	ORR, PFS, OS, DOR					[39]
IMpower 133	3	PT-DC +/-ATEZO	Advanced SCLC	OS, PFS, DOR, safety					[62]

## Malignant mesothelioma

Malignant mesothelioma is a deadly malignancy leading to the deaths of 2497 people in the USA in 2013. More than 80% of patients have clear asbestos exposure as the etiology. Five-year survival rates are estimated to be as low as 8%. Treatment typically involves surgery, radiation, and chemotherapy, but immunotherapy shows early but promising results [40]. A recent study which evaluated 170 malignant pleural mesotheliomas with IHC, ISH, and next generation and sanger sequencing demonstrated that significant fraction of tumors were positive for PD-1 and PD-L1 expression [41]. As described below, several completed and ongoing trials are looking at the efficacy of immunotherapy in mesothelioma.

Tremelimumab (CTLA-4 inhibitor) was evaluated in a phase 2 trial (DETERMINE) versus placebo in pretreated, malignant mesothelioma. Its primary endpoint was OS, and secondary endpoints included PFS, ORR, DCR, DOR, and safety. Unfortunately, there was no difference in OS between tremelimumab and placebo at 7.7 versus 7.3 months (HR = 0.92, 95% CI, 0.76–1.12, *p* = 0.408) [42].

Avelumab, a PD-L1 inhibitor, is being evaluated in a phase 1b study (JAVELIN) in pretreated, unresectable malignant mesothelioma patients. At interim analysis, 53 patients were evaluated at a median of 46 weeks. PFS rate at 24 weeks was 38.4% (95% CI, 23.3–53.4). 35.9% of the tumors were PD-L1+, and ORR was 14.3% in PD-L1+ tumors versus 8.0% in PD-L1− tumors. Median PFS was 17.1 weeks (95% CI, 5.4+) in PD-L1+ tumors versus 7.4 weeks (95% CI, 6.0-30.1) in PD-L1− tumors [43].

NIBIT-MESO1 is a phase 2 study that will evaluate durvalumab 20 mg/kg every 4 weeks in combination with tremelimumab 1 mg/kg every 4 weeks in first-line and second-line therapy for malignant pleural and peritoneal mesothelioma. Patients will be evaluated with a primary endpoint of irORR, and secondary endpoints include irDCR, irPFS, OS, DCR, PFS, and safety [44]. In summary, immunotherapy as a novel treatment modality for mesothelioma is promising but still early (see Table 5).

**Table 5:** Selected immunotherapy trials in malignant mesothelioma

Trial name	Phase	Therapy	mOS	1-year OS	DOR	mPFS (weeks)	1-year PFS	mORR (%)	Ref
DETERMINE	2b	Tremelimumab	7.7	NR	NR	NR	NR	NR	[42]
		Placebo	7.3	NR	NR	NR	NR	NR	
JAVELIN	1	Avelumab PD-L1+	NR	NR	NR	17.1	NR	14.3	[43]
		Avelumab PD-L1-	NR	NR	NR	7.4	NR	8.0	
Ongoing trial name	Phase	Therapy	Setting	Endpoints (starting with primary)					
NIBIT-MESO-1	2	Durvalumab + tremelimumab	Unresectable malignant mesothelioma	ORR, DCR, PFS, OS (by PD-L1%), and safety.					[44]

## Novel combinations

Only a subset of NSCLC patients respond to checkpoint blockade therapy; therefore, new combinations of therapy have been proposed to increase response rates and efficacy. Based on preclinical studies, several potential targets appear to be good candidates for inhibition. CD3 and CD8 are cell surface markers typically seen with T cells. PD-1 interacts with both PD-L1 and PD-L2 to downregulate T cell activation. CTLA-4 binds to CD80 or CD86 on APCs to decrease antigen presentation. IDO-1 is an enzyme that can deplete tryptophan leading to the decreased growth of T cells. B7-H4 is a surface protein that can negatively regulate T cells when interacting with them (Fig. 1). A number of novel combinations are currently being examined.

Increased levels of indoleamine 2,3-dioxygenase (IDO) can serve as a tumor derived immunosuppression mechanism via the increased metabolism of tryptophan to kynurenine. In addition, stage III NSCLC patients were evaluated before and after chemoradiation and patients with an elevated kynurenine/tryptophan ratio had worse OS (HR 1.25, 95% CI 1.01–1.56, *p* = 0.04) suggesting that IDO activity is a possible immune escape mechanism [45]. Therefore, IDO has been and continues to be evaluated as a potential target. The IDO inhibitor, indoximod, is being evaluated in a phase 1 setting alone and has been shown to be safe and efficacious in pretreated, advanced solid tumors. Out of the 48 patients on study, 10 patients had NSCLC. Hypophysitis was the main toxicity that was noted [46]. Indoximod is also being evaluated in combination with checkpoint inhibition therapy including nivolumab, pembrolizumab, and ipilimumab. The early phase 1/2 study will evaluate ORR in advanced melanoma. This combination is also being evaluated in other disease states along with NSCLC [47].

Nivolumab is being evaluated with an allogeneic whole-cell vaccine called viagenpumatucel-L or HS-110. This vaccine will be selected for adenocarcinoma tumor antigens and transfected to secrete gp96-Ig to the patient’s antigen presenting cells (APCs) leading to increased cytotoxic CD8+ TILs. Pretreated, advanced NSCLC patients will be stratified according to TIL findings (low TIL ≤10% CD8+ T cells versus high TIL >10% CD8+ T cells). The primary endpoints will include safety and then ORR with plans for biopsies at baseline and week 10 along with PD-L1 staining [48].

Pembrolizumab is being evaluated in combination with concurrent chemoradiation for stage III NSCLC. The primary endpoint is distant recurrence, and secondary endpoints include PFS, OS, and toxicity. During early evaluation, only one grade 3 urinary toxicity was reported [49].

Phase 1b/2 trials are evaluating pembrolizumab in combination with entinostat (a histone deacetylase inhibitor) in pretreated, advanced NSCLC. In animal models, blocking histone deacetylation with entinostat has been shown to decrease the activity of Tregs and suppressor T cells leading to increased T cell activation [50]. Early results show one grade 3 elevation in alkaline phosphatase and bilirubin concerning for mild hepatitis, and stable disease was seen in three out of six evaluated patients [51].

Pembrolizumab is being evaluated in early phase trials in combination with oral azacitadine in pretreated, advanced NSCLC. Hypomethylating agents such as azacitadine can lead to epigenetic changes that are proposed to lead to increased tumor immunogenicity and more responsive to immunotherapy. Patients will be stratified according to histology, with a primary endpoint of PFS, and secondary endpoints including DCR, OS, ORR, safety, and pharmacokinetics. Further exploratory endpoints will include PD-L1 expression, TILs, gene expression, and DNA methylation analysis [52].

IPI-549, a PI3K-gamma inhibitor, is under evaluation in multiple tumor types, including NSCLC, as monotherapy and in combination with pembrolizumab. PI3K-gamma has been shown to increase the immunosuppressive effects of myeloid cells in the tumor microenvironment, and inhibition of this molecule has shown decreased tumor growth in preclinical studies. This effect has been increased when IPI-549 was used in combination with checkpoint inhibition. A phase 1/1b study will help determine the MTD, PK, and safety and will further assess the efficacy in multiple tumor types including NSCLC [53].

Durvalumab is being evaluated in combination with bavituximab in pretreated, advanced NSCLC patients. Bavituximab is an inhibitor of phosphatidylserine which is an immunosuppressive molecule expressed on tumor cells and exosomes in the tumor microenvironment. Bavituximab has shown improvement in median OS in nonsquamous, pretreated, advanced NSCLC patients with combination with docetaxel over control (11.7 versus 7.3 months). Early phase trials will evaluate bavituximab in combination with durvalumab until progression or toxicity, with a primary endpoint of ORR, and secondary endpoints of PFS, OS, and safety [54].

## Resistance mechanisms

Duration of response to PD-1/PD-L1 inhibition is notably longer compared to cytotoxic regimens. However, some patients eventually progress and the etiology of resistance is an active area of research. Interestingly, small studies utilizing whole-exome sequencing in patients who initially responded to PD-1 checkpoint inhibition and then progressed found mutations involving interferon pathway genes. Loss of function mutations and truncating mutations were seen in JAK1, JAK2, and B2M proteins associated with the interferon signaling pathways. Further studies will be needed to verify these resistance mechanisms to checkpoint inhibition in lung cancer, but these findings suggest future therapeutic targets for patients who progress on checkpoint inhibition [55].

Increased Tregs and DCs in the tumor environment may be responsible for acquired resistance and provide another therapeutic target to prevent or overcome resistance. Tregs can be identified with the cell surface markers Foxp3, CD25, CD357, lymphocyte-activation gene 3 (LAG3), CTLA-4, and low CD127. Forkhead box protein 3 (Foxp3) are crucial in the immunosuppressive activity of suppressor T cells or Tregs within the lung cancer tumor microenvironment (TME). Foxp3 is a transcription factor that is upregulated in TILs and tumor cells and coveys a negative prognostic factor in the lung cancer and maybe a future target for resistant tumors. LAG3 is also a co-inhibitory molecule on TILs, Tregs, DCs, and NK cells that dampens T cell activation via its binding to MHC II receptors, making it another possible therapeutic target after resistance. T cell immunoglobulin and mucin domain-3-containing molecule 3 (TIM3) is a cell surface protein typically seen on DCs that interact with Galectin-9 on T cells leading to inhibition of the T cell response. TIM-3 expression can be seen on TILs, and its interaction with galectin-9 on Tregs or tumor cells can lead to T cell inhibition (Fig. 1). Increased TIM-3 expression has been seen as a marker of poor prognosis but may also provide an alternative checkpoint target for therapy after PD-1 failure [56, 57].

## Conclusions

Checkpoint blockade immunotherapy has revolutionized the treatment of lung cancer. PD-1/PD-L1 inhibitors such as nivolumab and pembrolizumab have shown improved efficacy and longer duration of response compared to standard-of-care chemotherapy (docetaxel). Treatment with nivolumab provided benefit in patients with advanced squamous cell carcinoma regardless of PD-L1 status while the efficacy of nivolumab is more pronounced in the PD-L1 positive, nonsquamous population compared to the PD-L1 negative, nonsquamous population. Pembrolizumab shows benefit in conjunction with its companion diagnostic for PD-L1 positive patients regardless of histology [58]. Atezolizumab is a PD-L1 inhibitor but has shown similar efficacy as its predecessors and benefit over standard-of-care chemotherapy in the pretreated setting.

Given the promising findings in the pretreated setting, checkpoint blockade therapy is being evaluated in the first-line setting also. The phase III study of nivolumab did not meet its primary endpoint of PFS while the study of pembrolizumab did. This is possibly because of the more stringent PD-L1 expression cutoff in the pembrolizumab trial (50%) compared to the nivolumab trial (5%). This has led to the FDA approval of pembrolizumab in the first-line setting for tumors with >50% PD-L1 positivity and established a new standard of care. Anti-PD-L1 agents are also being tested as monotherapy as well. While ipilimumab alone has not shown significant benefit, combination with nivolumab is still undergoing evaluation in the treatment-naïve setting but is promising based on the preliminary data. Combination therapy with pembrolizumab or atezolizumab and chemotherapy is still early, and finding the best histology and PD-L1 expression population will be beneficial. Furthermore, these agents are now being examined in the neoadjuvant and adjuvant settings where they may lead to a significant increase in lung cancer survival.

The benefit of checkpoint inhibition does not appear to be limited to NSCLC as promising results have been observed in extensive stage SCLC with nivolumab monotherapy and in combination with ipilimumab. Additional studies with anti PD-1/PD-L1 will define the true potential of these agents in SCLC. Trials for checkpoint blockade in malignant mesothelioma are ongoing but include therapies such as nivolumab, pembrolizumab, tremelimumab, avelumab, and durvalumab mostly in the pretreated setting.

Newer combinations are also being evaluated such as pembrolizumab plus ipilimumab in the pretreated NSCLC population or durvalumab plus tremelimumab in the treatment-naïve population. IDO inhibition with indoximod will be evaluated in various treatment settings alone and in combination with PD-1/PD-L1 inhibition. Pembrolizumab will be evaluation in the stage III setting with chemoradiation along with azacitadine, a PIK3-gamma inhibitor and an HDAC inhibitor in the pretreated, advanced population. Nivolumab will be evaluated in combination with a whole-cell vaccine, and durvalumab will be evaluated in combination with a phosphatidylserine inhibitor in the pretreated, advanced population.

The future for these agents as monotherapy and in combination with novel agents appears bright in lung cancer. However, there are many unanswered questions in regard to the proper use of the agents including the duration of use for these agents [59], which biomarker(s) will be predictive for response or toxicity, what leads to acquired resistance to these agent,s and which combinations will be the most effective in overcoming and preventing resistance? Over the next several years, it is likely that significant progress will be made in addressing many of these questions.

## Abbreviations

APC: Antigen presenting cells
ATEZO: Atezolizumab
CTLA-4: Cytotoxic T-lymphocyte-associated protein 4
DC: Dendritic cells
DOC: Docetaxel
DOR: Duration of response
HR: Hazard ratio
IHC: Immunohistochemistry
IPI: Ipilimumab
NIVO: Nivolumab
NONSQ: Nonsquamous
NSCLC: Non-small cell lung cancer
ORR: Overall response rate
OS: Overall survival
PD-1: Programmed death 1
PD-L1: Programmed death ligand 1
PEMBRO: Pembrolizumab
PFS: Progression-free survival
PK: Pharmacokinetics
SCLC: Small cell lung cancer
SQ: Squamous
TIL: Tumor-infiltrating lymphocyte
TrAE: Treatment-related adverse event
Treg: Regulatory T cell
